# Are Osteomeatal Complex Variations Related to Nasolacrimal Canal Morphometry?

**DOI:** 10.22038/IJORL.2021.52612.2784

**Published:** 2022-01

**Authors:** Leila Khojastepour, Sonia Dokohaki, Maryam Paknahad

**Affiliations:** 1 *Department of Oral and Maxillofacial Radiology, School of Dentistry, Shiraz University of Medical Sciences, Shiraz, Iran.*; 2 *Department of Oral and Maxillofacial Radiology, School of Dentistry, Iran University of Medical Sciences, Tehran, Iran.*; 3 *Oral and Dental Disease Research Center, Department of Oral and Maxillofacial Radiology, School of Dentistry, Shiraz University of Medical Sciences, Shiraz, Iran.*

**Keywords:** Cone-beam computed tomography, Morphometry, Nasolacrimal canal, Osteomeatal complex variation

## Abstract

**Introduction:**

Due to the close anatomic relationship between paranasal structures and NLC, the morphometric measure of the nasolacrimal canal (NLC) could be affected by the osteomeatal complex (OMC) anatomical variations. The present study aimed to assess the effect of OMC variations on the NLC morphometric features using cone-beam computed tomography (CBCT).

**Materials and Methods:**

This cross-sectional study consisted of CBCT images of 150 subjects in the case group with at least one OMC variation and 40 cases in the control group without any OMC variation within the age range of 18-50 years. The presence of the OMC variations, including agger nasi, nasal septum deviation, concha bullosa, Haller cells, paradoxical middle turbinate, and pneumatization of the uncinate process, was evaluated in each patient. The NLC morphometric measurements were performed and compared between the case and control groups.

**Results:**

The middle anteroposterior diameter and middle sectional area of NCL were significantly higher in patients with OMC variations, as compared to that in the control group. The NLC volume was significantly higher in patients with agger nasi, nasal septum deviation, concha bullosa, and pneumatization of the uncinate process, as compared to that in the control group. Nonetheless, no significant difference in NLC angulation with the nasal floor or Frankfurt horizontal plane was observed in the presence of each OMC variation.

**Conclusions:**

As evidenced by the obtained results, a higher volume of the canal was revealed in the presence of some of the OMC variations. Therefore, it can be suggested that OMC variations cannot be a predisposing factor in cases with primary acquired nasolacrimal duct obstruction.

## Introduction

The nasolacrimal canal (NLC) which opens at the inferior meatus of the nose (1) is a bony canal formed by indentations in the inferior nasal conchae, maxilla, and lacrimal bone. The anatomical characteristics of NLC, such as morphology and diameters, have been considered in several cadaveric and radiographic studies as a key factor in the development of primary acquired nasolacrimal duct obstruction (PANDO) (2-4). Although PANDO seems to be a common clinical entity in ophthalmology, detailed endoscopic examination and preoperative paranasal sinus computed tomography (CT) disclosed the possible role of nasal and paranasal structures adjacent to NLC in the etiology of NLC obstruction (5-8). 

Osteomeatal complex (OMC) is the final drainage pathway of the frontal, maxillary, and anterior ethmoidal air cells(9). It contains the maxillary sinus ostium, ethmoidal infundibulum, anterior ethmoid cells, and frontal recess(10). Haller cells, agger nasi cells, concha bullosa, paradoxical middle concha, enlarged bulla ethmoidalis, deviated nasal septum, and uncinate process variations are among the OMC anatomical variations. These variations may adversely affect the mucociliary clearance, resulting in sinusitis. (11, 12). Due to the close anatomic relationship between paranasal structures and NLC, the morphometric measure of NLC could be affected by the osteomeatal complex anatomical variations.

Preoperative CT is mandatory according to the recommendations of the working committee on the head and neck diagnostics of the German Radiological Society for sinus surgery (13). It especially provides high-resolution images and reliable morphometric information about various bony structures, such as NLC (14). In recent years, however, cone-beam computed tomography (CBCT) which offers better spatial resolution (smaller voxel size) with lower radiation doses has also become widely available (14). 

Multiple studies have assessed the co-occurrence and possible role of sinonasal anomalies in PANDO patients using CT or endoscopic examination and reported controversial results (5,8,15-17). To the best of our knowledge, only one study directly evaluated the relationship between concha bullosa and nasal septum deviation with the diameter of the nasolacrimal duct in CT images (18). In light of the aforementioned issues, the present study aimed to evaluate the effects of all OMC variations on the NLD morphometric features using CBCT.

## Materials and Methods

This cross-sectional study was approved by the institutional ethics committee. Among 996 paranasal CBCT images of the patients who were referred to the Oral and Maxillofacial Radiology Department of Shiraz Dental School between January 2018 to January 2020, 150 cases (75 males and 75 females) with at least one OMC variation, including Agger nasi (AN), Nasal septum deviation (NSD), Concha bullosa (CB), Haller cells (HC), Paradoxical middle turbinate (PMT), and pneumatization of the uncinate process (Pneumatized UP), were selected as the case group. Moreover, 40 cases (15 males and 25 females) without any OMC variations were selected as the control group. The subjects were within the age range of 18-50 years. Participants with a previous history of sinus tumor or surgery, sinonasal polyposis, and maxillofacial trauma, as well as those under 18 years old, were excluded from the study. As errors in patient positioning could result in inaccurate measurements, images with faulty patient orientations (such as head tilt) were also ruled out. Written consent was obtained at the time of radiographic examination from all the patients or their guardians for probable use of their anonymous information in future studies.

All the images were acquired using a New Tom VGi evo CBCT unit (QR SRL Co., Verona, Italy) with the following specifications: 75-110 kV, 1-32 mA, pulsed mode, Focal Spot 0.3 mm, amorphous silicon flat panel, scan time 15-25 s, and emission time 0.9-6 s. All CBCT images were taken in a standard voxel size (300 µm) in fields of view (FOV 16×16 cm). Image reconstruction and measurements were performed using the propriety New Tom software (NNT viewer, version 9.2). The measurements were performed by an oral and maxillofacial radiologist in a darkened room. The adjustment in density and contrast of the images was made, if necessary, for better assessment and measurement procedures. The slices were reconstructed with a slice thickness and slice interval of 1 mm. 

**Fig 1:**
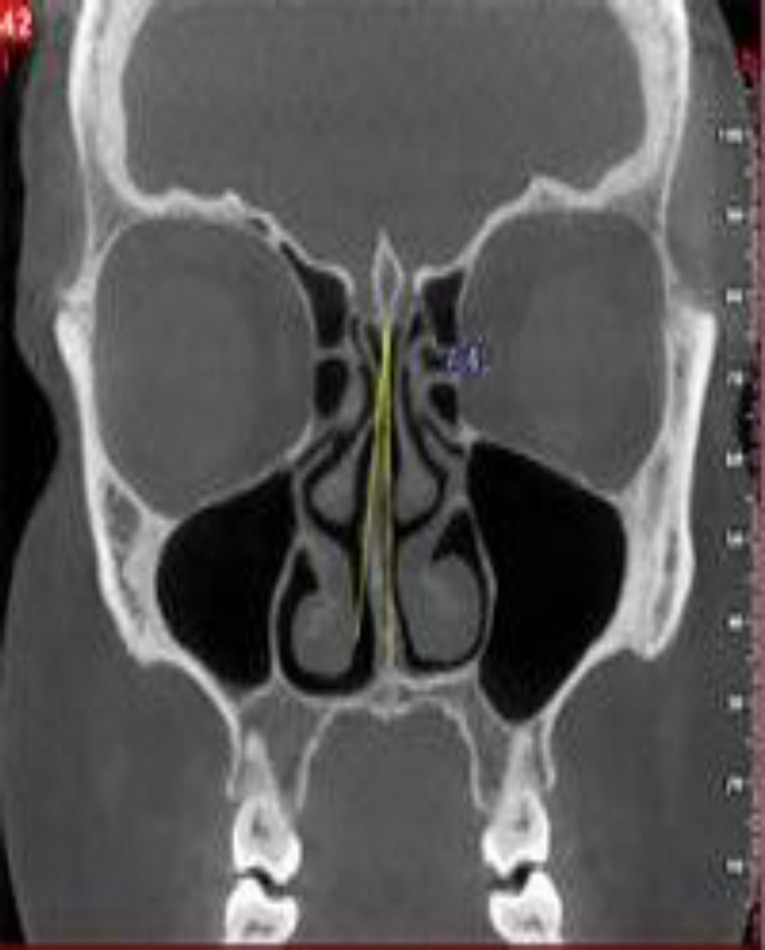
Measurement of the NSD angle in a sample case on the coronal cross-section

The presence of the following osteomeatal complex variations was evaluated in each patient: AN, NSD, CB, HC, PMT, and Pneumatized UP. The side and location of NSD (anterior, posterior, and middle) were determined and recorded based on the axial cross-section at the level of the inferior turbinate. The NSD angle was measured between the most deviated point of the septum and the midline on the coronal cross-sections (16) (Figure 1).

**Fig 2:**
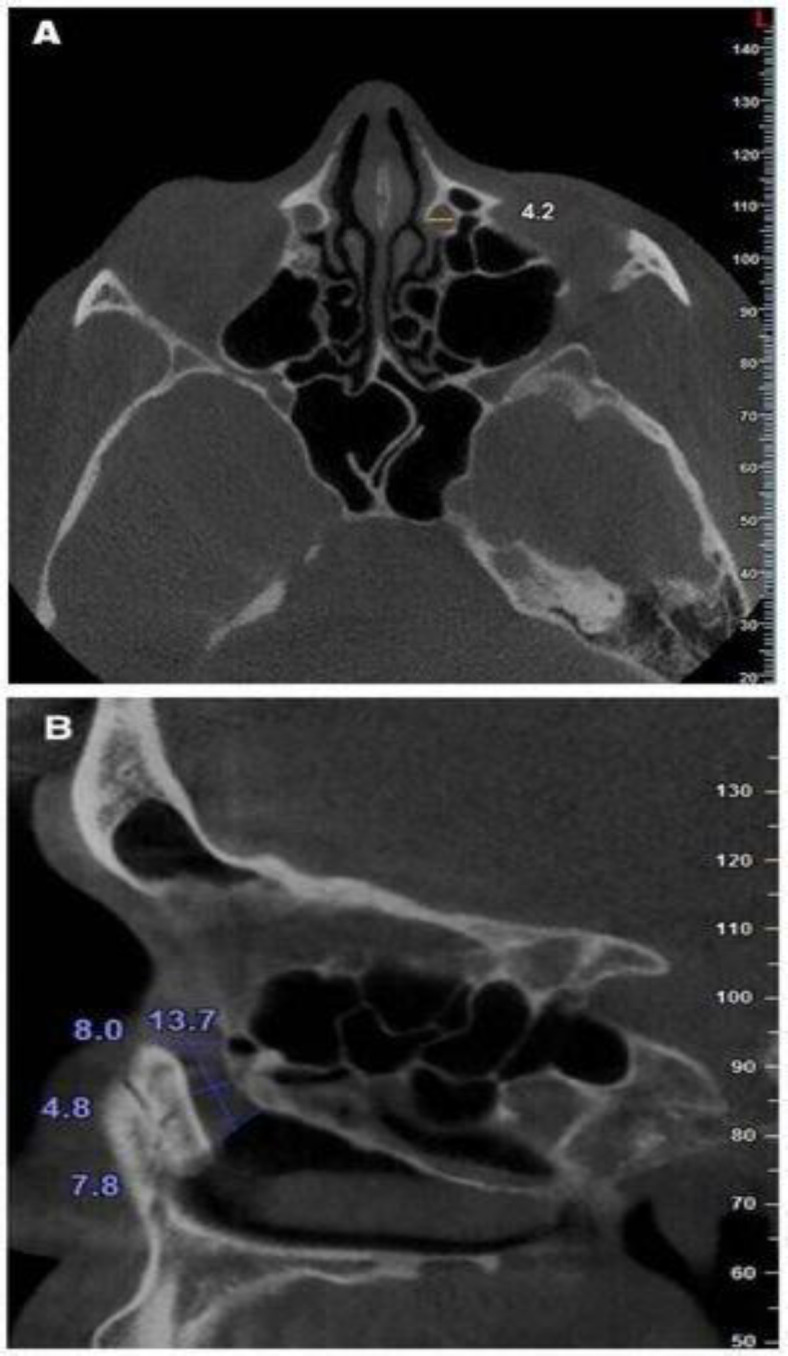
A) Measurement of the NLC transverse diameter on the axial image. B) Measurements of the NLC length and the NLC anteroposterior diameters at the proximal, middle, and distal ends on the sagittal image

**Fig 3:**
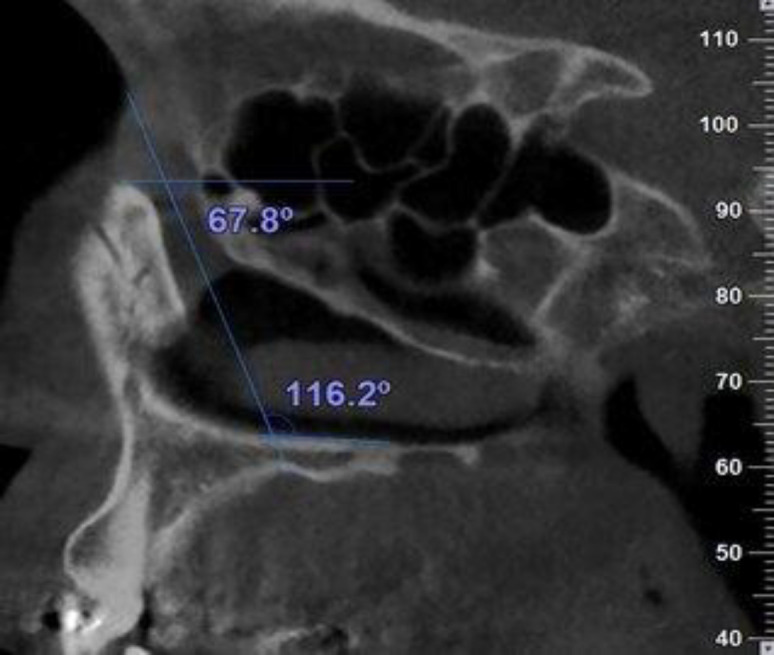
Measurement of the NLC angulation with the NF and FH on the sagittal image

The measurements of NLC included the following items: the transverse diameter of the NLC at the proximal (entrance), middle, and distal ends of the canal on the axial section (14) (Figure 2-A), the anteroposterior diameter of the NLC at the proximal (entrance), middle, and distal ends of the canal on the sagittal section (19) (Figure 2-B), the NLC length as the distance between the midpoints of the line that crossed the upper and lower anterior and posterior walls of the NLC on the sagittal section (20) (Figure 2-B), the angle between the long axis of the NLC and nasal floor (NF) on the sagittal section (21) (Figure 3), the angle between the long axis of the NLC and Frankfurt horizontal plane (FH) on the sagittal section (14) (Figure 3).

The sectional areas at the entrance, middle, and distal ends of the NLC were calculated based on the ellipse cross-sectional area by multiplying the half of the transverse diameter by the half of the anteroposterior diameter by π (3.14). The volume of the NLC was calculated based on the elliptical cylinder volume by multiplying the average cross-sectional area by the length. The total number of different OMC variations in each patient was also recorded. It was used to evaluate the possible correlations between NLC morphometric features and the coincidence of multiple OMC variations. The CBCT images, with a two-week interval between data recording phases, were evaluated again by the same observer to assess the significance of any errors during measurements. The CBCT images were evaluated by another maxillofacial radiologist. Intra- and inter-observer agreements were assessed using the intra-class correlation coefficient (ICC) and Kappa index for quantitative and qualitative variables, respectively.

***Statistical Analysis***

The data were analyzed in SPSS software (version 22.0) (SPSS Inc., IBM Corporation, NY, USA). The independent t-test was employed to compare the mean scores of age and nasolacrimal duct dimensions between the cases and controls. Moreover, the Chi-square test was used to compare the two groups in terms of gender. In addition, the one-way analysis of variance (ANOVA) with Tukey's multiple comparison tests was used to assess the relationship between the side of deviation and NLC volume in patients with NSD. The correlation between morphometric features of NLC and the total number of OMC variations in each patient was evaluated by Spearman Correlation. The significance level was considered 0.05.

## Results

The ICC and kappa indices indicated acceptable agreement between the observers (all ICCs>0.85 and all Kappa values>0.75). There were no significant differences in age (P=0.66) and gender (P=0.15) between the case and control groups. The demographic characteristics of the study groups are presented in Table 1.

**Table 1:** Demographic characteristics of the study groups

**Parameters**		**Case (n=150)**	**Control (n=40)**
Age, Mean ± SD		32.1±7.4	31.5±9.5
Gender	Male (%)	75 (50%)	15 (37.5%)
	Female (%)	75 (50%)	25 (62.5%)
NSD, N (%)		150 (100%)	NA
CB, N (%)		102 (68%)	NA
PMT, N (%)		40 (26.7%)	NA
AN, N (%)		142 (94.7%)	NA
UP, N (%)		41 (27.3%)	NA
HC, N (%)		30 (20%)	NA
		

The mean scores of NLC morphometric measurements for the case and control groups are illustrated in Table 2. The transverse and anteroposterior diameters, as well as the sectional areas of NLC, were measured at the entrance, middle, and distal ends of NLC. The anteroposterior dimension at the middle part of NLC was significantly higher in patients with OMC variations (P=0. 02). 

The middle sectional area was also higher in the case group (P=0.01). There was no significant difference between the mean score of NLC length in the case and control groups. The mean volume of NLC in patients with OMC variations was significantly greater in the case group, as compared to that in the controls (P=0.005). The mean angle between NLC and NF or FH was not statistically different between the groups.

**Table 2:** Mean scores of the NLC diameter, length, sectional area, volume, and angulation in case and control groups

**Measured NLC Parameters**	**Case group Mean ± SD**	**Control group Mean ± SD**	**P-value**
Transverse diameter upper(mm)	4.76±0.91	4.69±.86	0. 53
Transverse diameter middle(mm)	4.41± .98	4.30±.69	0.22
Transverse diameter Lower(mm)	4.71±0.98	4.78± 1.07	0.54
Anteroposterior diameter upper(mm)	6.91±1.29	6.62±1.17	0.07
Anteroposterior diameter middle(mm)	5.64±1.33	5.26±1.13	0.02
Anteroposterior diameter lower(mm)	7.35±1.50	7.09±1.26	0.14
Length(mm)	14.54±2.57	14.03±2.53	0.11
Sectional area upper(mm^2^)	26.18±8.37	24.69±7.44	0.14
Sectional area middle(mm^2^)	20.23±8.73	18.14±6.38	0.01
Sectional area lower(mm^2^)	27.87±10.64	27.13±9.53	0.57
Total volume (mm^3^)	359.89±130.70	323.46±90.90	0.005
NLC angulation with NF (degree)	114.09±9.59	113.77± 9.07	0.78
NLC angulation with FH (degree)	71.51±7.32	72.29± 6.75	0.39


The NLC volume was significantly higher in patients with NSD (anterior, middle, and posterior), as compared to that in the control group (Independent t-test, P=0.001, 0.01, and 0.004, respectively). 

Furthermore, the contralateral side of the deviation had higher NLC volume in patients with NSD (anterior, middle, and posterior), in comparison with that in the control group (ANOVA, P=0.017, 0.024, and 0.02, respectively). No statistically significant differences were observed between the NSD (anterior, middle, and posterior) and the NLC angulation with NF (Independent t-test, P=0.88, 0.78, 0.33, respectively) or FH (Independent t-test, P=0.36, 0.43, 0.55, respectively) between the case and control groups.

The mean scores of NSD angle in the anterior, middle, and posterior parts were obtained at 14.97±4.90, 10.92±3.88, and 12.51±3.98, respectively. The correlation of the mean angle of NSD (anterior, middle, and posterior) and the NLC volume and NLC angulations with NF and FH are demonstrated in Table 3. No statistically significant difference was found between the mean angle of NSD (anterior, middle, and posterior) and the NLC volume or NLC angulations with NF and FH. The relationship of other OMC variations with NLC volume and angulation is displayed in Table 4. 

**Table 3:** Correlation between the NLC measured parameters and NSD angulation in the anterior, middle, and posterior

**Side of NSD deviation**	**NLC Parameters**	**P-value**		
		**Anterior**	**Middle**	**Posterior**
Same	Total V	0.669	0.104	0.634
	Angle NF	0.750	0.509	0.473
	Angle FH	0.144	0.464	0.353
Contralateral	Total V	0.164	0.106	0.645
	Angle NF	0.489	0.109	0.134
	Angle FH	0.443	0.114	0.173

**Table 4:** Comparison of the NLC volume and angulation between case and control groups in the presence of each OMC variations

**Measured parameters**	**OMC variables**	**Case group**	**Control group**	**p-value**
		**mean ± SD**	**mean ± SD**	
Total V	AN	356.32±130.65	323.46±90.90	0.03
	CB	363.21±130.78	323.46±90.90	0.01
	PMT	351.66±129.48	323.46±90.90	0.11
	HC	359.42±119.44	323.46±90.90	0.054
	Pneumatized UP	367.85±131.82	323.46±90.90	0.01
Angle NF	AN	113.81±9.49	113.77 ±9.07	0.97
	CB	114.44±9.73	113.77±9.07	0.59
	PMT	113.98±11.58	113.77 ±9.07	0.89
	HC	112.78±9.27	113.77 ±9.07	0.52
	Pneumatized UP	112.57 ±9.93	113.77 ±9.07	0.42
Angle FH	AN	71.39± 7.40	72.29 ± 6.75	0.32
	CB	71.95±7.30	72.29 ±6.75	0.71
	PMT	72.40±8.72	72.29 ±6.75	0.92
	HC	72.00±6.94	72.29 ±6.75	0.80
	Pneumatized UP	71.49±9.24	72.29 ±6.75	0.53


The NLC volume was significantly higher in the presence of AN, CB, and pneumatized UP, as compared to that in the controls. Nonetheless, no significant difference was observed in NLC angulations in the presence of each OMC variation between the control and case groups. Spearman rank correlation test was used to assess the correlation between the NLC morphometric features and the total number of the OMC variations in each patient. Out of 150 patients, 7 (4.67%) cases had the maximum number of five OMC variations. It was followed by 18 (12%) patients with four, 68 (45.30%) patients with three, and 43(28.7%) patients with two OMC variations, respectively.

A number of 14 (9.33%) patients had only a single OMC variation. No significant correlation was found between the number of OMC variations and the measured parameters of NLC, including upper transverse diameter (P=0.059), middle transverse diameter (P=0.08), lower transverse diameter (P=0.32), upper anteroposterior diameter (P=0. 92), middle anteroposterior diameter (P=0.81), lower anteroposterior diameter (P=0.66), length (P=0.48), upper sectional area (P=0.23), middle sectional area (P=0.24), lower sectional area (P=0.94), total volume (P=0.84), NLC angulation with NF (P=0.68), and NLC angulation with FH (P=0.85).

## Discussion

Dacryocystorhinostomy is the treatment of choice for distal obstruction of the lacrimal system (22). The close relationship between the lacrimal system and the nasal cavity made endonasal surgical treatment of lower lacrimal disorders very popular among otorhinolaryngologists (22). The NLC is prone to damage during Dacryocystorhinostomy in probing of stenosis of the nasolacrimal duct, nasolacrimal complex fractures, or endoscopic medial maxillectomy (23-26). Therefore, detailed knowledge of the anatomical structure of the NLC is necessary to reduce surgery-related incidence of complications and mortality. Moreover, several studies have suggested that the morphology of the NLC could be a contributory factor in PANDO development (27, 28). Nasal and paranasal anatomic structures are in close association with NLC. As a result of this anatomic intimacy, several studies have reported that the nasal and paranasal sinus pathology often plays a major role in the etiology of NLC obstruction (5, 6). 

The present study assessed the effect of OMC variations on the NLC morphometric features based on CBCT images. Previous studies investigated the co-occurrence and the possible role of sinonasal anomalies on the morphometric features of NLC in PANDO patients (5, 8, 15-17). To the best of our knowledge, there is only one study in the literature conducted by Sirik et al. who investigated the relationship between OMC variations and the diameter of the nasolacrimal duct in the paranasal sinus CT of non-PANDO patients (18). Nevertheless, the authors only evaluated the effect of CB and NSD on the diameter of the NLC. This study assessed the relationship between all OMC variations and different morphometric features of the NLC. 

In the present study, the NLC volume was significantly higher in patients with NSD (anterior, middle, and posterior), as compared to that in the control group. On the contrary, Sirik et al. figured out no statistically significant difference between NSD and the NLC diameter by analyzing 103 cases who underwent paranasal sinus CT (18). Recently, Dikici et al. evaluated the septal deviation in the axial and coronal planes of paranasal sinus CT and reported a statistically significant relationship between PANDO and the axial location of the septal deviation (17). Yazici et al. also investigated the side and localization of the NSD in 40 patients treated for unilateral PANDO by CT images. They detected no statistically significant difference in the anterior, middle, and posterior locations of NSD between the case and control groups. Nonetheless, the side of the NSD was correlated with that of the PANDO (16).

 A higher incidence of NSD was also reported by Kallman et al. in patients with nasolacrimal outflow obstruction (6). Gray et al. evaluated the patients with NLC obstruction and septal deformity and reported continued epiphora in every patient (100%) with a septal deformity on the affected side (29). Singth et al. noticed a significant increase in the incidence of nasal septal deviation in PANDO cases and the laterality of the septal deviation corresponded to the side of NLD obstruction in 90% of cases (7). This finding is consistent with those obtained by Taban et al. and Samarei et al. on patients with acquired nasolacrimal duct obstruction (8,30).

In contrast to the aforementioned studies, Habesoglu et al. revealed that the incidence of NSD was not statistically significant in patients with unilateral PANDO on paranasal CT examination (5). Since there was significantly higher NLC volume in patients with NSD than the control group, it can be suggested that NSD cannot be a predisposing factor for PANDO. 

In the current study, the angle of NSD had no significant effect on the volume or angulations of NLC with NF and FH. Yazici et al. reported that the angle of the NSD was not statistically significant between the PANDO patients and the control group (16). On the other hand, Dikici et al. identified a statistically significant relationship between PANDO and the angle of NSD in the axial sections of the paranasal sinus CT (17).

The volume of NLC was also increased significantly in the presence of AN, CB, and pneumatized UP in the present study. At the same time, Sirik et al. reported no statistically significant relationship between the CB and NLC diameter (18). In addition, in agreement with a study conducted by Kaplan et al. (15), Yazici et al. found no significant difference in the incidence of the CB and AN between the PANDO patients and control group (16). Moreover, Kallman et al. revealed that the difference in the incidence of CB was not statistically significant in patients with nasolacrimal outflow obstruction, compared to that in the controls (6).

 Habesoglu et al. reported that the incidence of PMT and AN was not statistically significant between the PANDO and non-PANDO sides (5). However, a significant increase was noticed in the incidence of CB on the obstructed side of the nasolacrimal duct (5). Dikici et al. indicated that the incidence of AN and CB in patients with PANDO was significantly less than that in others. Nonetheless, PMT was more common in the PANDO group (17). In agreement with the results of a study conducted by Behboudi et al. (2021), Samarei et al. observed a significant increase in the relative frequency of AN on the PANDO side of the cases, compared to that in the controls (30,31). According to the higher volume of NLC in the presence of AN, CB, and pneumatized UP, as well as no significant difference in NLC volume in the presence of other OMC variations between the control and case groups, it can be concluded the presence of these OMC variations can be just co-occurrence in PANDO cases. However, during external or endonasal DCR surgery in patients with primary NLD obstruction, the sinonasal pathologies, such as NSD, CB, AN, and PMT should be evaluated and corrected simultaneously since the success of the surgical outcome will be affected (32).In line with the results of the research performed by Shigeta et al. (3), in the current study, the middle of the NLC had a smaller diameter than the entrance and distal ends in both case and control groups. On the contrary, the smaller diameter of the upper end of the NLC, compared to the middle part and lower ends of NLC, was noted by Kolsuz et al. in pre-orthodontic evaluation CBCT (14). This disparity can be ascribed to racial differences. The NLC anteroposterior diameter at the middle part was the only measured parameter which significantly increased in patients with OMC variations (middle sectional area and NLC volume also increased consequently). This can be attributed to the limitation that OMC variations may be imposed on the mediolateral diameter, increasing the NLC dimension in the anteroposterior aspect. Multiple osteomeatal complex variations can occur more frequently than a single one (11). Therefore, the correlation between the maximum number of different OMC variations in each patient and NLC features were also evaluated, and no significant correlation was figured out. According to Behboudi, the presence of AN was not significantly correlated with the coexistence of CB and NSD as attributing factors for NLD obstruction (31). Along the same lines, Ramey et al. disclosed that the NLC morphometric features changed with aging (19). Therefore, the current study was conducted on a limited range of subjects from the age of 18-50 years old for minimizing the effect of this variable on the results. Unequal male to female ratio and lower number of patients in the control group could be regarded as the notable limitations of the present study. It is, however, inevitable in this study group. Based on previous studies, males are more prone to trauma (33) and have deviated nasal septum, compared to females (34). 

## Conclusion

According to the higher volume of NLC in the presence of AN, CB, and pneumatized UP, as well as no significant difference in NLC volume in the presence of other OMC variations between the control and case groups, it can be concluded that the presence of these OMC variations can be just co-occurrence in PANDO cases. Nonetheless, prior to DCR surgery in patients with primary NLD obstruction, the OMC variations should be evaluated and corrected simultaneously since the success of the surgical outcome will be affected.
